# Supplementary material for: A MD Simulation Prediction for Regulation of N-Terminal Modification on Binding of CD47 to CD172a in a Force-Dependent Manner
Source: Molecules. 2023 May 22;28(10):4224. doi: 10.3390/molecules28104224 (PMC10221095; doi:10.3390/molecules28104224)
Supplement: Supplementary file 1 [file molecules-28-04224-s001.zip › Supplementary Material.pdf]

Supplementary Materials

A MD Simulation Prediction for Regulation of N-terminal Modification on Binding of CD47 to CD172a in a force-dependent manner

Yang Zhao, Liping Fang, Pei Guo, Ying Fang * and Jianhua Wu *

Institute of Biomechanics/School of Biology and Biological Engineering, South China University of Technology, Guangzhou 510006, CHINA;

* Correspondence: yfang@scut.edu.cn (Y, F); wujianhua@scut.edu.cn (J, W)

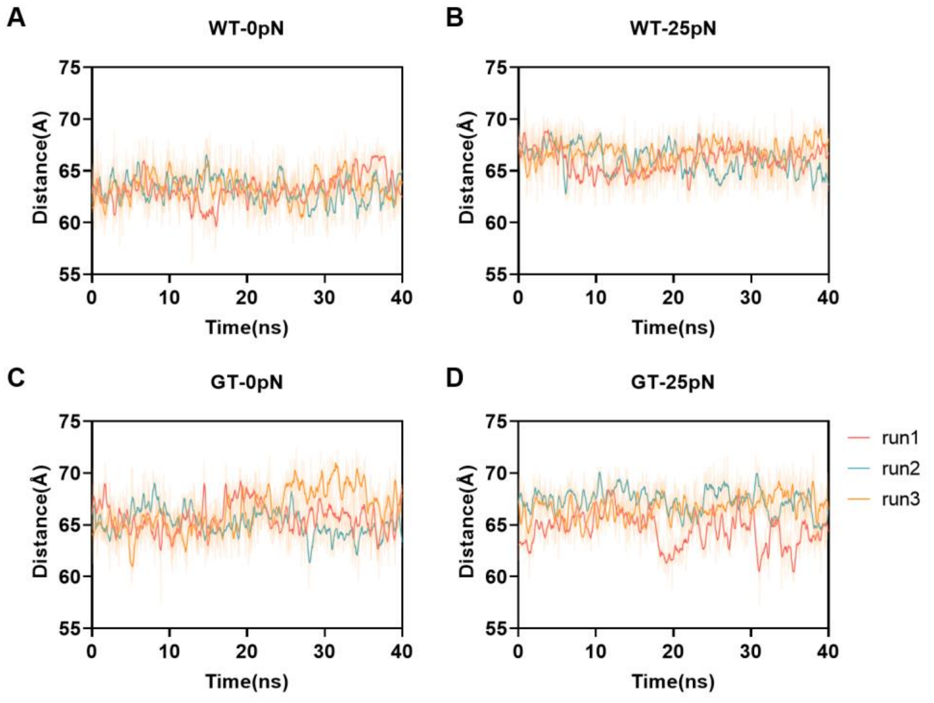

Figure S1. Time-courses of distance between Cα atom of VAL115 in CD47 and Cα atom of ALA115 in CD172. All data were sampled from "force-clamp" SMD runs of 40 ns thrice for WT-SS (A and B) and GT-SS (C and D) system at static state or under tensile force of 25 pN.

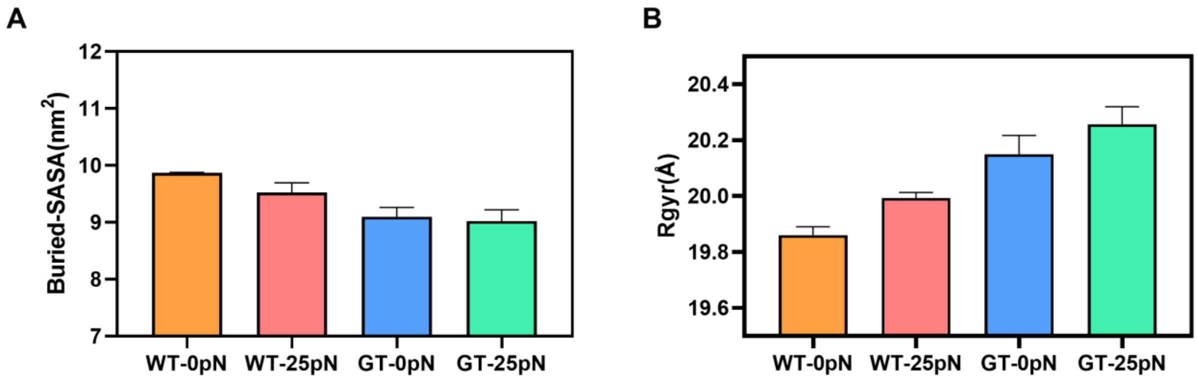

Figure S2. Tension-induced structural change of CD172a. The plots of the Buried-SASA **(A)** and the Rgyr **(B)** over thrice 40 ns "force-clamp" SMD runs for four systems, the WT-SS and GT-SS systems at static state or under tensile force of 25 pN.
